# Systematic analysis reveals molecular characteristics of ERG-negative prostate cancer

**DOI:** 10.1038/s41598-018-30325-9

**Published:** 2018-08-27

**Authors:** Qingyu Xiao, Yidi Sun, Albert Dobi, Shiv Srivastava, Wendy Wang, Sudhir Srivastava, Yuan Ji, Jun Hou, Guo-Ping Zhao, Yixue Li, Hong Li

**Affiliations:** 1 CAS Key Laboratory of Computational Biology, CAS-MPG Partner Institute for Computational Biology, Shanghai Institute of Nutrition and Health, Shanghai Institutes for Biological Sciences, University of Chinese Academy of Sciences, Chinese Academy of Sciences, Shanghai, P. R. China; 2 Center for Prostate Disease Research, Department of Surgery, Uniformed Services University of the Health Sciences and Walter Reed National Military Medical Center, Bethesda, MD USA; 3 Cancer Biomarkers Research Group, Division of Cancer Prevention, National Cancer Institute, Bethesda, MD USA; 4 Department of Pathology, Zhongshan Hospital, Fudan University, Shanghai, China

## Abstract

The *TMPRSS2:ERG* gene fusion is the most prevalent early driver gene activation in prostate cancers of European ancestry, while the fusion frequency is much lower in Africans and Asians. The genomic characteristics and mechanisms for patients lacking *ERG* fusion are still unclear. In this study, we systematically compared the characteristics of gene fusions, somatic mutations, copy number alterations and gene expression signatures between 201 *ERG* fusion positive and 296 ERG fusion negative prostate cancer samples. Both common and group-specific genomic alterations were observed, suggesting shared and different mechanisms of carcinogenesis in prostate cancer samples with or without *ERG* fusion. The genomic alteration patterns detected in *ERG*-negative group showed similarities with 77.5% of tumor samples of African American patients. These results emphasize that genomic and gene expression features of the *ERG*-negative group may provide a reference for populations with lower *ERG* fusion frequency. While the overall expression patterns were comparable between *ERG*-negative and *ERG*-positive tumors, we found that genomic alterations could affect the same pathway through distinct genes in the same pathway in both groups of tumor types. Altogether, the genomic and molecular characteristics revealed in our study may provide new opportunities for molecular stratification of *ERG*-negative prostate cancers.

## Introduction

Prostate cancer is the second most commonly diagnosed cancer type in men globally and the fifth leading cause of cancer death, accounting for 6.6% of death among men^1^. Significant efforts have been made to characterize recurrent genomic alterations in prostate cancers, which may be potential driver events^2–5^. The overall mutation burden in prostate cancer is relatively low (0.3–2 non-synonymous somatic mutations per megabase) compared to other cancer types^2,6,7^. The most common genomic alteration is the fusion of 5′-UTR of *TMPRSS2* (21q22) with 3′-end of ETS family members, such as *ERG* (21q22), *ETV1* (7p21), *ETV4* (17q21), or *ETV5* (3q27)^8–11^. Significantly mutated genes include *SPOP*, *FOXA1*, *TP53*, *MED12*, and *CDKN1B*^2,5,12^. In addition to somatic mutations, somatic copy number alterations (SCNA) are recurrently seen in prostate cancer, including the amplification of chromosome 7 and 8q (affecting the *MYC* locus), and the focal deletion of chromosome 1q42, 3p13 (*FOXP1*), 4p15, 6q12–22 (*MAP3K7*), 8p, 13q, 16q, 17p (*TP53*), 18q12, and 21q22.3 (*TMPRSS2-ERG* fusion)^5,7,12,13^. However, there is still a large proportion of prostate cancer genomes that remains to be evaluated^5,14,15^.

Further studies confirmed that the *TMPRSS2*-*ERG* fusion is caused by an interstitial deletion on chromosome 21 or by a chromosomal translocation. These genomic rearrangements results in the overexpression of the *ERG* oncogene and ERG oncoprotein^16,17^. A variety of biological processes and pathways including cell invasion, Androgen receptor (AR) signaling, Transforming growth factor beta 1 (TGF-β) signaling have been implicated in *ERG* dysregulation^18–22^. *ERG* oncogenic activation is an early causal event in prostate cancer^23–25^. In some reports *TMPRSS2-ERG* fusion is positively correlated with advanced tumor stage, high Gleason score, and worse survival^17,26–30^. While some studies did not found significant association between *ERG* fusion and disease progression^26,31–34^, numerous studies reported positive correlation of ERG-negative prostate tumor type with disease progression^35–37^.

Since *TMPRSS2*-*ERG* fusion is a dominant molecular subtype in prostate cancer in European descents, it provides opportunities for targeted cancer therapy. Along these lines, direct and indirect *ERG* targeted therapeutic approaches are being developed^38–40^. Patients harboring ERG oncoprotein positive tumors are more likely to benefit from ERG targeted therapy. However, the frequency of *TMPRSS2-ERG* fusion significantly varies in different ethnic groups^41^. African American (20%~30%) and Asian (less than 20%) has much lower fusion frequency compared to Caucasian (~50%)^42–44^. In contrast to *ERG* fusion positive tumors, the genomic characteristics are not yet clear for the *ERG* fusion negative tumor type^45^. Therefore, identification of driver events in *ERG*-negative prostate cancer is important for understanding the mechanism of tumorigenesis.

In this study, we systematically explored the genomic and molecular differences of gene fusions, somatic mutations, SCNAs, gene expression signatures and dysregulation of pathways in prostate tumors with or without *ERG* fusion using publicly available data. Our results provide new insights into the molecular landscape highlighting specific mechanisms of prostate tumorigenesis.

## Results

### Data sources and the relationship between *ERG* fusion, deletion, and expression

**Figure 1:**
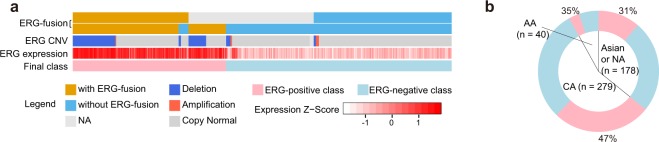
Classification of prostate cancer samples based on ERG fusion. **(a)** The relationship among ERG fusion, copy number variation, ERG mRNA expression and the final sample group. The top two rows show ERG fusion status based on the evidences from TCGA research article (333 samples) and TFGDP database respectively (See Method). **(b)** The proportion of ERG-positive and ERG-negative samples in different ethnic groups. AA: African American; CA: Caucasian American. Asian is not shown because of the small sample size.

We collected the *ERG* fusion status information from two prostate cancer genome studies and compared the relationship among *ERG* fusion, deletion and expression^5,46^. The two datasets were highly consistent, except for 13 samples where the fusion status was unclear in the genomic data (Fig. 1a). We checked the *ERG* expression in these 13 samples and found significantly higher expression compared with *ERG* fusion negative samples (t.test, p-value = 0.001), indicating *ERG* may be activated in these 13 samples. Therefore, we assigned a sample into the ERG-positive group if its ERG fusion was detected in either study. As a result, we identified 201 *ERG*-positive samples and 296 *ERG*-negative samples for subsequent analysis (Supplementary Table 1). Also, we used *ERG* gene expression to verify the genomic classification of samples. Since, *ERG* fusion could result from either translocation or deletion at 21q22.3^16^, we found that 40.8% *ERG*-positive samples harbored *ERG* deletion.

Clinical characteristics of the *ERG*-positive and *ERG*-negative groups are summarized in Table 1. Although, patients with higher Gleason score (4 + 3 or 8–10) were more frequently found in the *ERG*-negative group, biochemical recurrence-free survival of patients showed no difference between the two groups (Supplementary Fig. 1, p-value = 0.29, Log-rank test). The TCGA prostate cancer cohort contained 279 Caucasian American (CA), 40 African American (AA), 5 Asian men and 173 without known ancestry. The proportion of ERG-positive samples in CA was higher than that in AA (47% vs. 35%, Fig. 1b), which is in accordance with previous studies (Supplementary Table 2). Like TCGA, most of the previous studies focused on patients of European ancestry. Indeed, more studies are needed for African and Asian patients that harbor mostly ERG-negative prostate cancers.

**Table 1:** Distribution of clinical variables stratified by ERG status (n = 497).

		Overall	ERG-positive (n = 201)	ERG-negative (n = 296)	p-value
Clinical characteristics	Age(median)	61.5 (42–78)	61 (42–76)	62 (44–78)	9.29E-03
	PSA(mean)	1.74	1.14	2.18	4.31E-01
Gleason Score	< = 6	44	19	25	8.20E-01
	3 + 4	146	70	76	**3**.**59E-02**
	4 + 3, 8–10	306	111	195	**2**.**13E-02**
Pathologic Stage	pT2a/b	23	14	9	6.78E-02
	pT2c	164	64	100	7.23E-01
	pT3a	158	65	93	9.06E-01
	pT3b	135	54	81	9.84E-01
	pT4	10	3	7	7.47E-01
PSA Recurrence	yes	58	23	35	1
	no	371	146	215	1
	Not available	68	22	46	—
Ethnicity	Caucasian	279	131	148	**1**.**14E-03**
	African descent	40	14	26	5.73E-01
	Asian	5	2	3	1
	Not available	173	54	119	—

### Common and specific genomic alterations in *ERG*-positive and *ERG*-negative prostate cancers

#### Gene fusions

**Figure 2:**
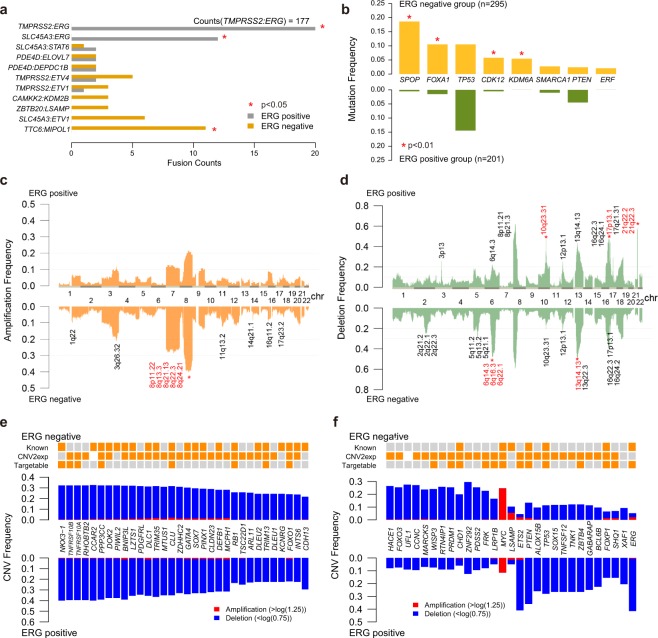
Comparision of the genomic characteristics between ERG-positive and ERG-negative groups. **(a)** The number of gene fusions in the two groups. Fusions present in more than two patients are shown. Red stars: fusions with significantly different frequency (p < 0.05, fisher test). **(b)** The frequency of significantly mutated genes in the two groups. Genes with significantly different mutation frequency in the two groups are marked with red stars. (p < 0.01, chi-squared test). The frequency of copy number amplification **(c)** and deletion **(d)** in two groups. Significantly altered cytobands separately detected in each group are annotated in black. Recurrently altered cytobands with significantly different frequency between the two groups are indicated in red. (chi-squared test, p < 0.01; frequency > 30%). Common **(e)** and group-specific **(f)** SCNA genes.

Consistent with previous studies^47,48^, in *ERG*-positive group, the most frequent fusion partner of *ERG* in our study was *TMPRSS2* (94.1%), and the second was the *SLC45A3* gene (6.4%, located at 1q32.1, Fig. 2a). These two genes both have AR responsive promoter and share similar mechanisms in *ERG* overexpression^48^. As expected, significantly higher *ERG* expression was detected in samples harboring *SLC45A3:ERG* fusion compared with samples with non-detectable *ERG* fusion (pvalue = 5e-5, one-tailed t.test). Other two ETS-family members, *ETV1* and *ETV4*, show relatively high genomic rearrangement frequencies in *ERG*-negative group (4.7% and 2.7%, respectively). We found that the *LSAMP* gene that is frequently deleted in ERG-negative prostate tumors of African American men^49^, was often rearranged including fusion with *ZBTB20* specifically in the *ERG*-negative group. Moreover, tumor suppressor gene *MIPOL1* and *TTC6* fusion were also specifically detected in the *ERG*-negative group at notable frequency (3.7%, Fig. 2a). Recent study of 65 Chinese prostate cancer whole genomes also reported *TTC6:MIPOL1* fusion detected at 6.2% frequency^44^. Indeed, detection of *TTC6:MIPOL1* fusion may have potential implication for prostate cancers of non-European ancestry. In addition, ten of eleven recurrent gene fusions (detected at least in three samples) have been reported in other literatures. Thus prostate cancer genomic fusions detected in our study, as well as in other reports are more likely real than false positives (Supplementary Table 3).

#### Somatic mutations

We used MutSigCV to identify significantly mutated genes in the *ERG*-positive and *ERG*-negative groups respectively^50^. Only two genes, *TP53* and *PTEN*, were significantly mutated in *ERG*-positive group. By contrast, eight genes were significantly mutated in the *ERG*-negative group (Fig. 2b). In addition to known recurrently mutated genes *SPOP* and *FOXA1* which were reported to be mutually exclusive with *ERG* rearrangements^2,5^, we found that the mutation frequency of *CDK12* and *KDM6A* were significantly higher in the *ERG*-negative group (Fig. 2b, p-value = 1.18e-3 and 3.26e-4, respectively. Fisher.test).

#### Somatic copy number alterations

We applied the GISTIC algorithm to discern significant copy number alterations in the *ERG*-positive and *ERG*-negative groups^51^. First, we assessed the overall distribution of copy number alterations of all prostate cancer genomes in our study (Fig. 2c,d). Overall, deletions were more commonly than amplifications showing similar distribution in both *ERG*-positive and *ERG*-negative groups. Copy number alterations affected similar regions within the two groups, while deletion and amplification frequencies showed variations.

Twenty one amplified regions including chromosome 8q, 11q13, 14q21, 16q11, 1q22, 3q26 and 17q23, were recurrently altered in the *ERG*-negative group (Supplementary Table 4, residual q value < 0.05). The *ERG*-positive group harbored similar amplified regions, but did not reach statistical significance due to lower frequencies. Among the regions of copy number gains, chromosome 8q that includes the *MYC* oncogene exhibited a relatively high frequency (~40%). In another complex CNV region at 14q21.1 spanning *MIPOL1/FOXA1/TTC6* locus, the *MIPOL1:TTC6* gene fusion was detected. Moreover, we found several chromosome arm-level amplifications with significantly higher frequency in the *ERG*-negative tumors than in *ERG*-positives, including chromosome 8 (38.5% vs. 19%) and chromosome 7 (26.1% vs. 11.5%) (Fig. 2c).

Ten regions were commonly deleted in both *ERG*-positive and *ERG*-negative groups, including 6q14.3, 13q14.13, 10q23.31, 12p13.1, 5q11.2, 5q13.2, 17p13.1, and 16q22.3 (residual q < 0.05), which is consistent with previous reports^5,13^. Twenty two and twenty five copy number losses were detected only in the *ERG*-positive or in the *ERG*-negative group, respectively. Among these focal deleted regions, some showed significantly different frequency between the two groups. Similar to previous studies we also detected frequent deletions of 21q22 (*ERG*, *TMPRSS2*), 17p13.1 (*TP53*), and 10q23.31 (*PTEN*) in *ERG*-positive tumors, while 6q14.3 and 13q14.13 deletions were more frequent in *ERG*-negatives (Fig. 2d). Additionally, two novel regions, 6q16.3 (*HACE1*) and 6q22 (*FRK*) were deleted more frequently in the *ERG*-negative group.

To gain more insight into the functional effects of SCNA regions, we assessed the genomic defects of tumor suppressor genes (TSGs) and oncogenes (Supplementary Table 5). Thirty-two TSGs were recurrently altered with frequencies higher than 20% in both groups (Fig. 2e). Twenty-one (65.6%) of these genes were previously shown to play roles in the progression of prostate cancer. Other genes with high alteration frequencies need to be further defined. Thirteen TSGs and one oncogene showed significantly higher alteration frequency in the *ERG*-negative group, and another thirteen tumor suppressor genes and one oncogene showed significantly higher alteration frequency in ERG-positives (Fig. 2f). The candidate CNV genes found in TCGA dataset show comparable alteration frequency in an independent whole genome sequencing dataset, which includes 7 *ERG*-positive and 7 *ERG*-negative prostate tumors (Supplementary table 5, CPDR dataset). Among these group-specific SCNA genes, we found that ten genes were significantly associated with biochemical recurrence. In addition to previously reported disease progression related genes *TP53*, *PTEN* and *FOXP1*, we also found that an additional seven genes were associated with biochemical recurrence (Supplementary Fig. 2). Although *ERG* rearrangement status alone might not be a definitive marker for disease progression, our findings highlight a subset of genes associated with higher risk of disease progression (Overall prevalence: 44.87%). Furthermore, we found a group of tumor suppressor genes including *FRK*, *WISP3*, *PRDM1*, and *LRP1B* whose CNV and expression may indicate interactions with known drugs and therefore, are potentially actionable (Supplementary Fig. 3).

### Candidate genes associate with genomic alteration patterns in ERG-negative prostate tumors

**Figure 3:**
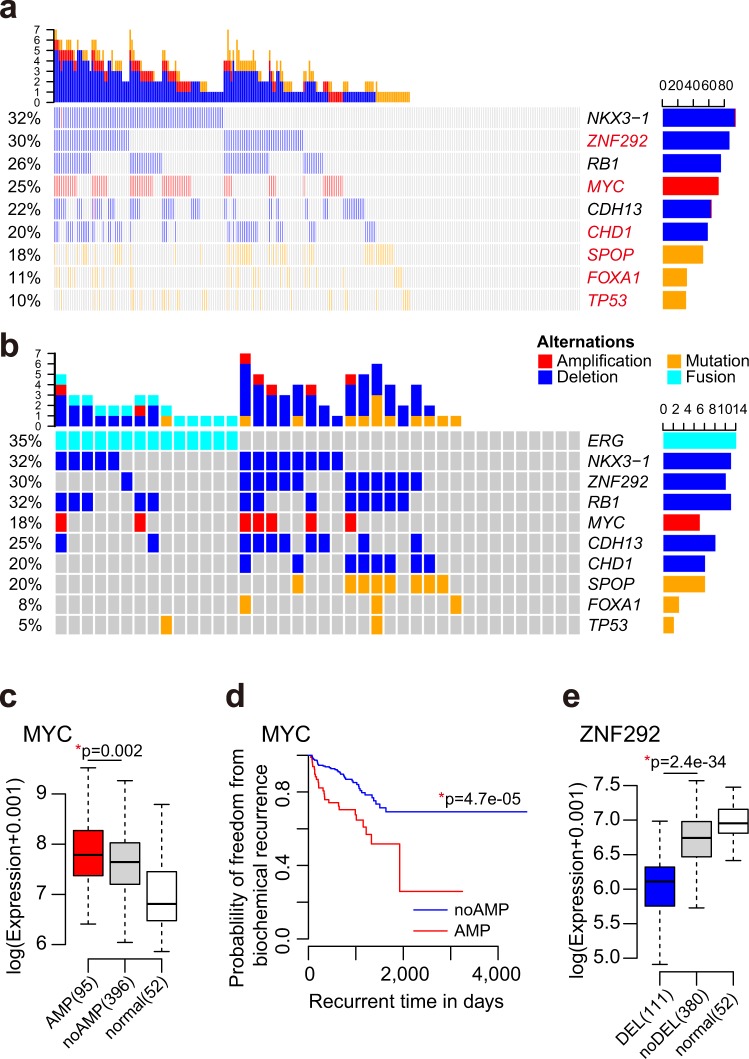
Genomic portraits of ERG-negative group and similar patterns in African Americans. **(a)** Alteration pattern of the most representative genes in ERG-negative group, covering 67.7% samples. Red: genes with significantly higher alteration frequency in ERG-negative group. **(b)** Similar patterns were found in 40 AA samples with the coverage of 77.5%. **(c)** MYC amplification is significantly correlated with expression (p = 0.002, t-test) **(d)** Kaplan–Meier plot of biochemical relapse-free survival probabilities of patients with and without MYC amplification (p = 4.7e-05, Log-rank test). **(e)** The expression of ZNF292 is significantly lower in samples with deletion (p < 2.2e-16, t-test).

Since we have characterized both common and group-specific genomic alterations with high frequency in *ERG*-positive and *ERG*-negative prostate tumors, we next examined the molecular portrait of the *ERG*-negative group based on the associated candidate genes. First, we combined the genomic alterations of gene fusions, somatic mutations and copy number alterations which occur recurrently in the *ERG*-negative group. Next, we removed the redundant alterations to find a subset of genes highly represented in the genomic alteration pattern of *ERG*-negative tumors. Nine representative genes have emerged from the analysis (Fig. 3a). Genomic alteration of one or more of these nine genes were detected in 67.7% of the *ERG*-negative group.

Since *ERG*-rearrangement are less frequent in prostate cancers of African descents, we explored whether candidate gene defects found in the *ERG*-negative group are present or absent in prostate cancers of AA men. As *ERG* is less frequent in prostate cancers of AA patients, we evaluated the alteration patterns of the nine genes characteristic to *ERG*-negative tumors in available datasets of 40 AA prostate tumor samples. We found that 77.5% AA tumors harbor at least one of the nine gene signatures associated with *ERG*-negative tumors indicating similar patterns between prostate cancers of AA patients and the overall genomic alteration pattern of ERG-negative tumors (Fig. 3b).

Among the nine representative genes, *NKX3-1*, *RB1*, and *CDH13* were commonly deleted in both *ERG*-positive and *ERG*-negative tumors. Other genes had significantly more alterations in *ERG*-negative samples. The oncogene *MYC* mRNA is up-regulated in tumor compared to normal. Tumors with *MYC* amplification show significantly higher expression of *MYC* gene and higher probability of disease progression than other patients (Fig. 3c,d). The Zinc finger transcription factor, *ZNF292* was shown to function as a tumor suppressor in gastric cancer, colorectal cancer, and chronic lymphocytic leukemia^52,53^. Deletion of ZNF292 in prostate cancer results in decreased expression (Fig. 3e), which may promote tumor development.

### Comparison of methylation and expression between *ERG*-positive and *ERG*-negative tumors

Since promoter hypermethylation is widely observed in multiple cancers, we investigated the hypermethylated sites in promoter regions (TSS200, TSS1500, 5’UTR and 1stExon) of genes with low mRNA expression (See Method). Compared to normal samples, 2191 CpG sites (694 genes) and 1871 CpG sites (645 genes) were hyper-methylated in *ERG*-positive and *ERG*-negative groups, respectively. Approximately 70% of them were overlapped between the two groups (Supplementary Fig. 4a). Direct comparison between two tumor groups indicated 51 hyper-methylated sites (31 genes) in *ERG*-negative and 14 hyper-methylated sites (8 genes) in *ERG*-positive tumors (Supplementary Fig. 4b). Therefore, the overall methylation profiles showed similarities between the two groups.

**Figure 4:**
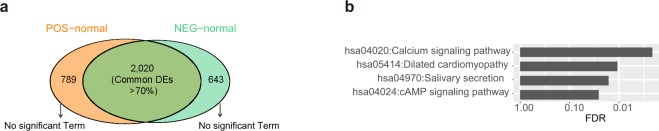
Gene expression in ERG-positive and ERG-negative groups. **(a)** Venn diagram for differentially expressed genes (DEs) detected in ERG-positive and ERG-negative groups respectively compared to normal samples. 2020 DEs (>70%) are common. **(b)** The enriched KEGG pathways for the common DEs (FDR < 0.05). No significantly terms were enriched for group-specific DEs.

We compared the expression profiles of *ERG*-positive, *ERG*-negative tumor and prostate tissue samples with morphologically normal appearance to identify differentially expressed genes among these three groups. A large proportion (>70%) of differentially expressed (DE) genes were common in the *ERG*-positive and negative groups (Fig. 4a). As expected, common DEs were significantly enriched in essential pathways like calcium signaling and cAMP signaling pathways (Fig. 4b). Common up-regulated genes were significantly enriched in cell cycle which is recurrently altered in cancer. However, no significant functional GO term was enriched for group-specific genes indicating comparable expression profiles between *ERG*-positive and *ERG*-negative prostate tumor types, despite in their differences in their dominant driver genomic alterations. These findings indicate that different genomic alternations may have similar effects on gene expression, resulting in similar phenotype.

### The impact of genomic alterations on pathway dysregulation in *ERG*-positive and *ERG*-negative prostate tumors

**Figure 5:**
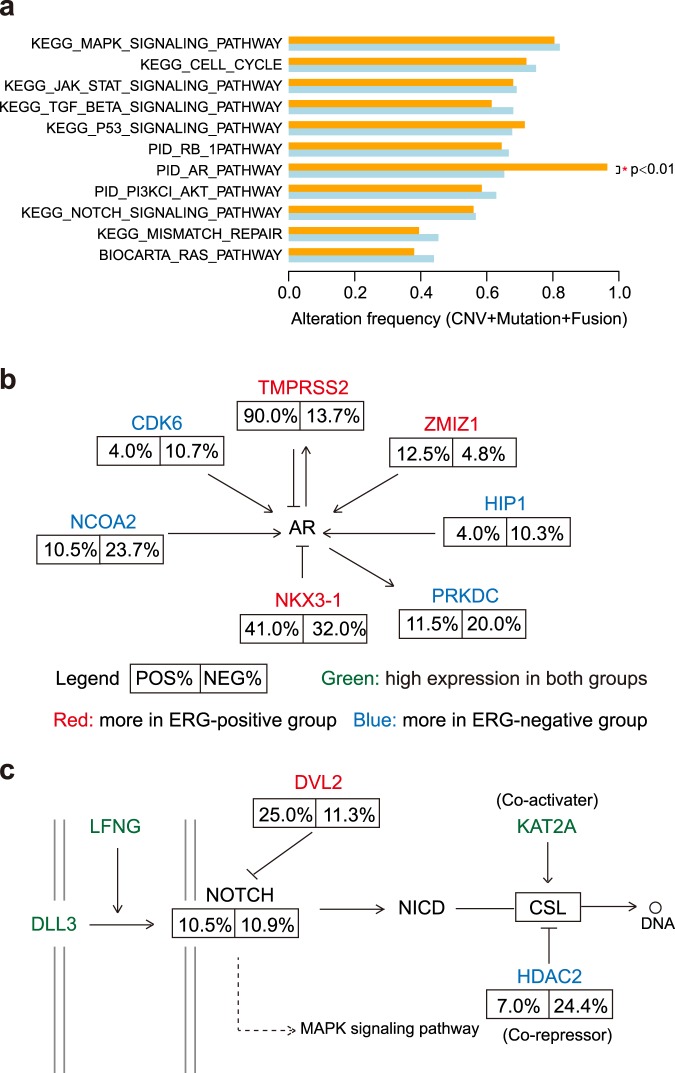
Genomic alterations show different preference on pathway dysregulation in ERG-positive and ERG-negative groups. **(a)** The overall alteration frequency on prostate cancer-related pathways. AR pathway show significant difference in two groups (p < 0.01). Genomic alterations in AR pathway **(b)** and NOTCH signaling pathway **(c)**. Genes with significantly different alteration frequency in the two groups are shown. Red: higher frequency in ERG-positive group. Blue: higher frequency in ERG-negative group. Green: high expression in both groups.

We selected eleven pathways either cancer-related or reported to be important in prostate cancer from Misgdb^54–56^. Next, we compared the frequency of CNV, somatic mutation and gene fusion of the *ERG*-positive and *ERG*-negative groups based on publicly available TCGA data. The male hormone axis (AR pathway) was the only node that altered significantly more frequently in *ERG*-positive group that is consistent with the AR regulation of *ERG* in the context of *TMPRSS2:ERG* fusion (Fig. 5a). However, there were still 65.3% of *ERG*-negative samples with AR pathway disruption, which were apparently affected by other genes in the AR pathway (Fig. 5b). For example, *CDK6* (10.7% *ERG*-negative vs. 4% ERG-positive), *NCOA2* (23.7% *ERG*-negative vs. 10.5% *ERG*-positive) and *PRKDC* (20.0% *ERG*-negative vs. 11.5% *ERG*-positive). Similarly, some component of NOTCH signaling pathway signatures had higher alteration frequency in the *ERG*-positive group (e.g., *DVL2*,11.3% *ERG*-negative vs. 25.0% *ERG*-positive) while *HDAC2* (24.4% *ERG*-negative vs. 7.0% *ERG*-positive) had higher alteration frequency in *ERG*-negative group (Fig. 5c). They both inhibit NOTCH signaling pathway but function at different contexts. Therefore, the observed prostate cancer genomic and expression alterations of different genes may affect the same pathway resulting in comparable expression profiles between *ERG*-positive and *ERG*-negative prostate tumor types.

## Discussion

Our study provides new insights into the molecular landscape of *ERG*-negative prostate cancers. Except for known alterations mutually exclusive with *ERG* rearrangements, such as mutation in *SPOP* and *FOXA1*, we found that gene fusion of *TTC6:MIPOL1* and somatic mutation on *CDK12* and *KDM6A* occurred more frequently in the *ERG*-negative group. Recurrent gene fusions and somatic mutations could explain only a subset of *ERG*-negative tumors, noting that more of these genes harbor somatic copy number alterations. Some of them are shared between the two groups of tumors, others occurred more frequently in one group over the other. In addition to confirm several previous studies, we found novel recurrent SCNA for *ERG*-negative prostate cancers, such as *ZNF292* deletion. In summary, the *ERG*-negative group was found more heterogeneous in our study.

When validated, the recurrently altered genes in specific patient groups may contribute to better tumor stratification and prognosis. Among these genes, *MYC* is a well-known oncogene that plays an important role in tumor progression. The amplification of *MYC* is frequently observed in numerous human cancers^57^. In this study, we found that *MYC* amplification frequency was significantly higher in the *ERG*-negative group. As expected, patients with tumors harboring *MYC* amplification show a strong association with poor outcome. Previous studies have reported that intact *CHD1* is required for *ERG* rearrangements in the process of tumor initiation and deletion of *CHD1* is mutually exclusive with ETS fusions^58^, that was consistently observed in our study. In addition to confirming known gene defects, we also identified several novel prostate cancer associated genes which may play important roles in the tumorigenesis of *ERG*-negative cancer type.

Our study highlights potentially actionable genes which may provide opportunities for target therapy of *ERG*-negative prostate tumors. These findings include the frequent deletion of the tumor suppressor gene *FRK* (6q22.1), a tyrosine-protein kinase that negatively regulates cell proliferation^59^, in *ERG*-negative group (22.3% vs. 8.0%). Decreased expression of *FRK* gene strongly correlated with its deletion. Moreover, FRK protein could interact with known drugs and may have potential application in clinical practice^60^. Other potentially druggable genes including *WISP3* (6q21), *LRP1B* (2q22.1), and *PRDM1* (6q21)^60,61^. In total, 21 (34.4%) genes in our candidate gene list have potential clinical relevance, covering 66.7% of *ERG* negative tumors.

Interestingly, we found that different gene alterations may result in similar expression change or pathway alteration. NOTCH signaling pathway is a typical example. Similar phenomenon has been observed in other cancer types. Taken Wnt signaling pathway as an example, *TP53*, *CTNNB1* and *AXIN1* are important elements in Wnt signaling network; *CTNNB1* is more frequently mutated in HCV-infected hepatocellular carcinoma (HCC)^62^, while the mutations of *TP53* and *AXIN1* are more frequent in HBV-infected HCC^63,64^, which indicated different viral etiologies might activate Wnt signaling in distinct ways.

Increasing number of studies reports race/ethnicity differences in cancer research. Due to the lack of large-scale omics study of African and Asian prostate cancer patients, directly comparisons among multiple races are challenging. Our focuse on the *ERG*-negative group could provide a reference for populations with low frequency of *ERG* positive tumor types. Nine representative genes were sufficient to classify into sub-categories 67.7% *ERG*-negative tumors that was consistently seen in 77.5% of prostate cancers of African American men. Our previous studies found that approximately 20% Chinese patients harbor *ERG*-positive tumors^41^. Therefore we are particularly interested in the frequently altered and targetable genes in the *ERG*-negative tumor type. The validation of the genomic alteration and expression of these genes in Chinese patients is warranted. Accumulating data on *ERG* negative prostate cancer will help to discover more disease progression associated and actionable driver genes. Additionally, further experimental assessments of the functional significance for recurrent genomic and gene expression alterations are also warranted.

Our study highlights new aspects of *ERG*-positive and *ERG*-negative prostate cancers at genomic, epigenetic, and expression levels. In this study, multi-omics data integration provided a methodological reference to prioritize candidate CNV genes and to evaluate the effects of overall alterations. The observed molecular differences on gene fusions, somatic mutations and copy number alterations between ERG-positive and ERG-negative prostate tumors suggest both common and distinct mechanisms of prostate tumorigenesis. Genes with recurrent alteration may act as potential drivers and contribute to patient stratification into distinct prognostic or therapeutic groups. These results will help experimental biologist and clinical doctors for further assessment of the functional significance of candidate genes. Together, our results provide new insights into prostate tumorigenesis further refining the sub-classes of *ERG*-negative and *ERG*-positive prostate tumor types.

## Methods

### Data collection

Somatic mutation (496 tumor samples), SCNA (492 tumor samples), methylation (497 tumor + 35 normal samples), and expression (497 tumor + 52 normal samples) data from TCGA primary prostate cancer cohort were used in this study^65^. Clinically actionable genes and the interactions between genes and drugs were retrieved from DGIdb (http://dgidb.org/)^60^.

### Patient group and ethnic information

Samples were stratified into ERG-positive and ERG-negative groups based on the combined ERG fusion evidences from TCGA research article (333 samples) and TFGDP database (http://www.tumorfusions.org/, 502 samples)^5,46^. A patient was assigned to ERG-positive group if its ERG fusion was detected in either study. For genome wide fusion analysis and statistics except for ERG fusion, data from TFGDP database was used. The ethnic information was collected from literature in which G. Petrovics *et al*. determine the ancestry of TCGA cohort by principal component analysis based on SNP genotype data^49^.

### Detection of significantly mutated genes and copy number alterations

We used MutSigCV (version: 1.2) to detect significantly mutated genes for ERG-positive and ERG-negative groups, respectively^50^. Chi-squared test and Fisher exact test (determined by theoretical frequencies and sample size) were used to test the significance of different alteration frequency between the two groups. GISTIC 2.0 (version 6.10) was used to identify genomic regions that are significantly amplified or deleted in ERG-positive and ERG-negative groups, respectively^51,66^. To find the common and specifically altered regions in the two groups, we divided the whole genome into consecutive bins (window length = 10 kb). For each bin, the SCNA status is determined by the SCNA status of majority of bases in it (that is, longer than 5 kb). For arm-level SCNA regions, the frequency was estimated by the median frequencies of all bins in that region.

Since the significant SCNA regions usually contained huge genes, we focused on the copy number alterations of tumor suppressor genes (TSGs) and oncogenes. We obtained 1217 TSGs and 232 oncogenes from TSGene Database (v2.0) and UniProtKB database (keyword:“Proto-oncogene [KW-0656]”)^67,68^. These genes were classified into two types based on the following filtering rules: 1) Common SCNA genes: high frequency (>20%) in both ERG-positive and ERG-negative groups; 2) Group-specific SCNA genes: TSGs (Oncogenes) whose deletion (amplification) frequencies were significantly different between two groups (P < 0.001) and the frequency difference was larger than 10%.

### Selection of representative genes for ERG-negative group

We used genes with recurrent SCNA (frequency >15%) or mutation (frequency >10%) as candidate feature genes for ERG-negative group. We defined a group of genes with higher priority: genes whose alteration frequency were significantly higher in ERG-negative group than that in ERG-positive group, genes which were targetable or had interaction with drugs, and genes whose copy number alteration was significantly correlated with expression. To remove genes with similar alteration pattern, we calculated Pearson correlation coefficient between genes and did unsupervised hierarchical clustering. For each cluster, we selected genes with the highest frequency or higher priority as the representative genes. At last, six CNV genes and three mutation genes were selected as final representative feature genes for ERG-negative group. OncoPrint was used to display the mutation landscape in ERG-negative group^69^.

### Independent validation dataset

We used an independent whole genome sequencing data (CPDR dataset) to validate the CNV candidate genes. The CPDR dataset including 7 ERG-positive and 7 ERG-negative prostate tumors. The Genomatix software suite/NGS Analysis (http://www.genomatix.de) was used for CNV calling.

### Differential expression analysis

We identified the differentially expressed genes among ERG-positive (n = 201), ERG-negative (n = 296) and normal samples (n = 52). Normalized read counts were used to detect differential expression genes with R package voom and limma^70^. Genes with P value < 0.05 and the absolute value of fold change (FC) > 2 were considered as differentially expressed.

### Differential methylation analysis

We identified the differentially methylated genes among ERG-positive (n = 201), ERG-negative (n = 296) and normal samples (n = 35) based on TCGA methylation data. Firstly, we removed the probes on X/Y/M chromosome or NA. Secondly, we found diff-methylated sites with t-test p < 0.01 and the absolute difference of beta value > 0.2. Thirdly, we selected diff-methylated sites on promoter region (TSS200, TSS1500, 5’UTR and 1stExon). Fourthly, we retained methylation sites negatively correlated with the corresponding gene expression in a cis-regulatory manner. Fifthly, we concentrated on hyper-methylated sites whose corresponding genes have significantly lower expression in tumor samples compared to normal samples. For the comparison between ERG-positive and ERG-negative group, genes hyper-methylated in either group were taken into account.

### Data availability

All data generated or analyzed during this study are included in this published article (and its Supplementary Information files).

## Electronic supplementary material

supplementary information

Supplementary Tables 1-4-5
