# Supplementary material for: Systematic analysis reveals molecular characteristics of ERG-negative prostate cancer
Source: Sci Rep. 2018 Aug 27;8:12868. doi: 10.1038/s41598-018-30325-9 (PMC6110738; doi:10.1038/s41598-018-30325-9)
Supplement: Supplementary file 1 — supplementary information [file 41598_2018_30325_MOESM1_ESM.pdf]

Systematic analysis reveals molecular characteristics of ERG-negative prostate cancer

Qingyu Xiao[1], Yidi Sun[1], Albert Dobi[2], Shiv Srivastava[2], Wendy Wang[3], Sudhir Srivastava[3], Yuan Ji[4], Jun Hou[4], Guo-Ping Zhao[1], Yixue Li[1]*, Hong Li[1]*

[1] Key Lab of Computational Biology, CAS-MPG Partner Institute for Computational Biology, Shanghai Institutes for Biological Sciences, University of Chinese Academy of Sciences, Chinese Academy of Sciences, Shanghai, P. R. China

[2] Center for Prostate Disease Research, Department of Surgery, Uniformed Services University of the Health Sciences and Walter Reed National Military Medical Center, Bethesda, MD, USA

[3] Cancer Biomarkers Research Group, Division of Cancer Prevention, National Cancer Institute, Bethesda, MD, USA

[4] Department of Pathology, Zhongshan Hospital, Fudan University, Shanghai, China

* Correspondence should be addressed to Hong Li and Yixue Li

Address: 320 Yueyang Road, Shanghai, P.R.China, 200031

Tel: 86-21-54920079

E-mail: lihong01@sibs.ac.cn (Hong Li); yxli@sibs.ac.cn (Yixue Li)

Supplementary Information

Supplementary Figure 1. Kaplan-Meier curve of biochemical recurrence-free survival in ERG-positive and ERG-negative groups.

Supplementary Figure 2. Kaplan-Meier plot for SCNA genes that associated with biochemical recurrence and differently altered in ERG-positive and ERG-negative groups.

Supplementary Figure 3. The relationship between CNV status and expression for clinically relevant genes.

Supplementary Figure 4. The differentially methylated sites and genes between ERG-positive and ERG-negative groups.

Supplementary Table 2. Previously published ERG fusion frequency in different races.

Supplementary Table 3. Recurrent gene fusions (detected in at least three samples) supported by other literatures.

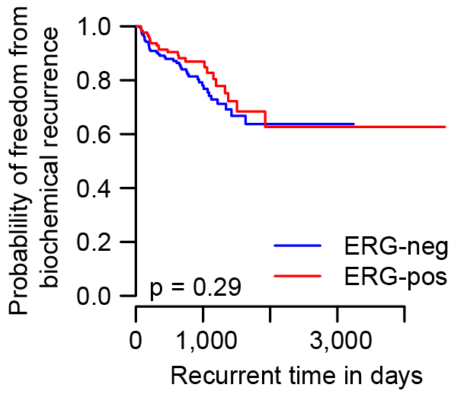

Supplementary Figure 1. Kaplan-Meier curve of biochemical recurrence-free survival in ERG-positive and ERG-negative groups. The results showed no difference between the two groups (p-value = 0.29, Log-rank test).

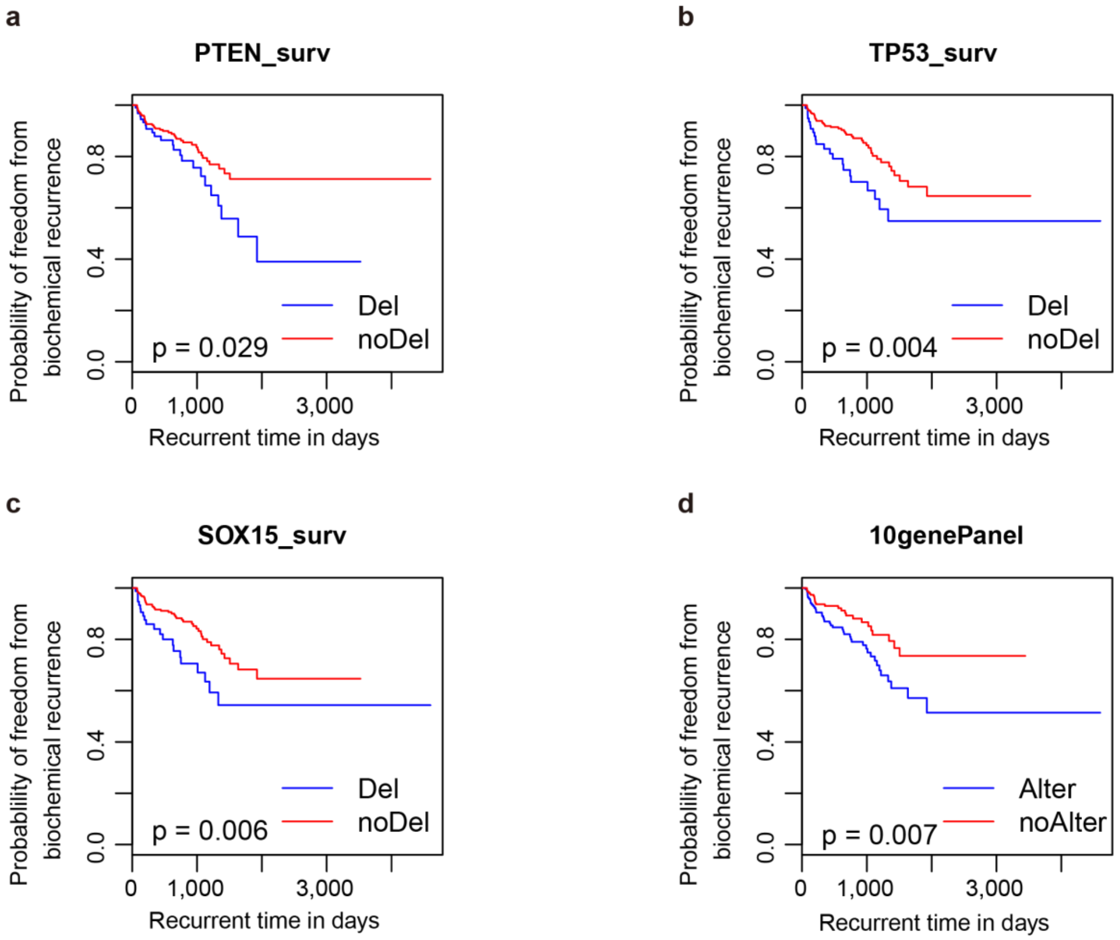

Supplementary Figure 2. Kaplan-Meier plot for SCNA genes that associated with biochemical recurrence and differently altered in ERG-positive and ERG-negative groups. *PTEN* deletion (a) and *TP53* deletion (b) are associated with relapse in accordance with previous studies. (c) Deletion of tumor suppressor gene *SOX15* is significantly associated with biochemical recurrence. (d) Ten SCNA genes could define a subgroup of patients with higher risk of recurrence (Overall prevalence: 44.87%).

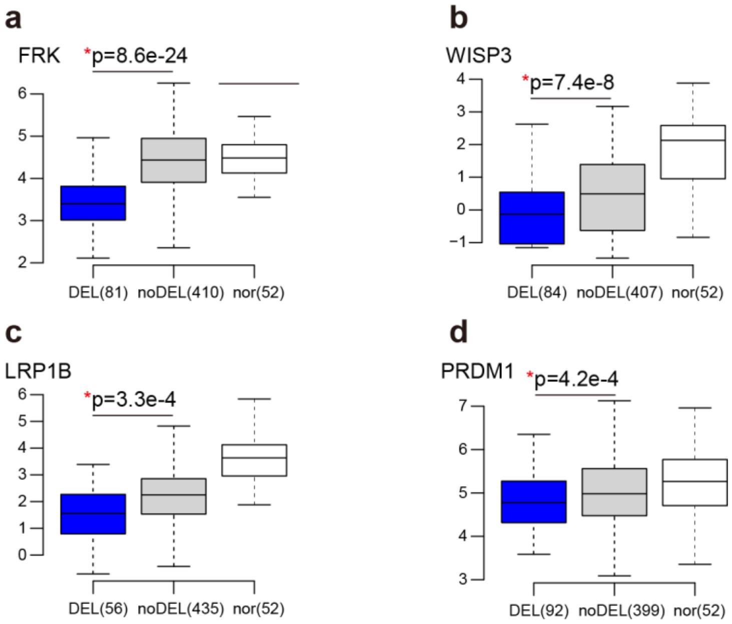

Supplementary Figure 3. The relationship between CNV status and expression for clinically relevant genes. Significant correlation was observed for *FRK* (a), *WISP3* (b), *LRP1B* (c), and *PRDM1* (d).

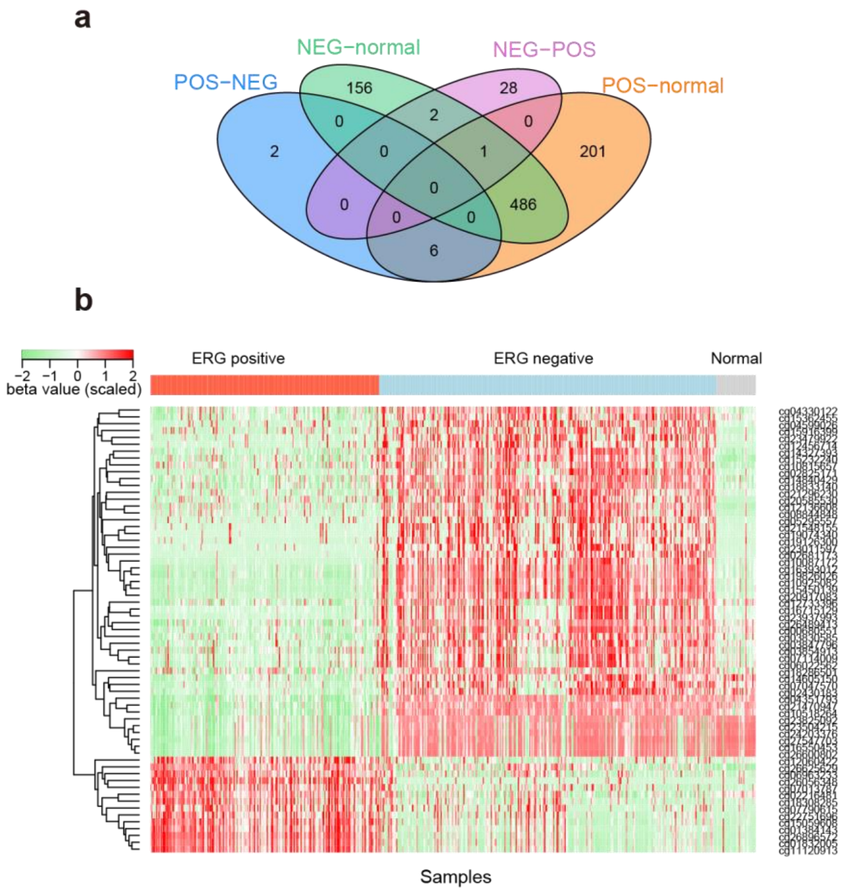

Supplementary Figure 4. The differentially methylated sites and genes between ERG-positive and ERG-negative groups. (a) Comparison of differential methylated genes among different groups. "POS-normal" ("NEG-normal") indicates the comparison between ERG-positive (ERG-negative) cancers with normal samples. "POS-NEG" and "NEG-POS" are the comparison between ERG-positive and ERG-negative groups. For any comparison, the figure showed genes that are hyper-methylated and lower expression in the first group. (b) The heatmap of scaled beta-value for group-specific hyper-methylated sites. 51 hyper-methylated sites (31 genes) in ERG-negative group and 14 hyper-methylated sites (8 genes) in ERG-positive group were selected by directly comparing ERG-positive and ERG-negative groups.

Supplementary Table 2. Previously published ERG fusion frequency in different races.

	CA	AA	Asian	method	sample size	reference	Year
**ERG fusion frequency**	51.5%	28.40%		immunohistochemistry using the 9FY mouse monoclonal anti-ERG antibody	443 AA and 696 CA	10.1158/1538-7445.AM2015-5277	2015
	41.90%	23.90%		expression	91 Caucasian American and 91 African American	Urology. 2012 Oct; 80(4): 749–753.	2012
	50%	31.3%	15.90%	multicolor interphase fluorescence in situ hybridization assay	42 Caucasians, 64 African-Americans, and 44 Japanese	10.1002/pros.21265	2010
	52%			FISH assay	134 samples (100 cancer and 34 benign) from North American cohorts	Clin Cancer Res. 2009 Jul 15; 15(14): 4706–4711.	2009
			6.20%	whole-genome and transcriptome sequencing	65 Chinese	http://dx.doi.org/10.1016/j.eururo.2017.08.027	2017
	European (54%) and North American (48%)		23%	meta-analysis	Asian 837; Europe: 4926; North America 3217	Cancer Epidemiol Biomarkers Prev. 2012 Sep; 21(9): 1497–1509.	2012
			28%	reverse-transcriptase polymerase chain reaction	194 Japanese prostate cancer	doi:10.1038/modpathol.2010.149	2010
			21.40%	RNAseq	14 Chinese prostate cancer samples	doi:10.1038/cr.2012.30	2012
			20.9%	FISH	254 prostate cancers in Korean patients	doi: 10.1016/j.urology.2010.06.010	2010

Supplementary Table 3. Recurrent gene fusions (detected in at least three samples) supported by other literatures.

Recurrent_Fusion*	counts_in_pos	counts_in_neg	Reported_in_literature	Reference (selected)
TMPRSS2:ERG	177	0	YES	[1] Nat Rev Cancer. 2008 Jul;8(7):497-511. [2] Neoplasia. 2008 Feb;10(2):177-88.
SLC45A3:ERG	12	0	YES	[1] Pathol. 2010 Apr;23(4):539-46.
SLC45A3:STAT6	2	1	NO	
PDE4D:ELOVL7	2	2	YES	[1] Cell. 2015 May 21; 161(5): 1215–1228.
PDE4D:DEPDC1B	2	2	YES	[1] Nat Biotechnol. 2015 Mar;33(3):306-12. (ovary cell line: RMG-I) [2] Cell. 2015 May 21; 161(5): 1215–1228.
TMPRSS2:ETV4	2	5	YES	[1] Cancer Res. 2006 Apr 1;66(7):3396-400. [2] J Mol Diagn. 2010 Sep;12(5):718-24.
TMPRSS2:ETV1	1	3	YES	[1] Science. 2005 Oct 28;310(5748):644-8. [2] Genes Chromosomes Cancer. 2006 Jul;45(7):717-9.
CAMKK2:KDM2B	0	3	YES	[1] Clin Cancer Res; 23(24); 7596–607.
ZBTB20:LSAMP	0	3	YES	[1] EBioMedicine. 2015 Oct 31;2(12):1957-64.
SLC45A3:ETV1	0	6	YES	[1] Nature. 2007 Aug 2;448(7153):595-9.
TTC6:MIPOL1	0	11	YES	[1] Int J Mol Sci. 2018 Feb 7;19(2). [2] Eur Urol. 2017 Sep 18. pii: S0302-2838(17)30720-0.

*Recurrent_Fusion: detected in at least three samples.
